# Mendelian randomization analyses support causal relationship between gut microbiota and childhood obesity

**DOI:** 10.3389/fped.2023.1229236

**Published:** 2023-08-01

**Authors:** Qi Li, Jiawei Gao, Jiashun Luo, Dihui Lin, Xinrui Wu

**Affiliations:** ^1^ School of Medicine, Jishou University, Jishou, China; ^2^ Department for Infectious Disease Control and Prevention, Xiangxi Center for Disease Control and Prevention, Jishou, China

**Keywords:** childhood obesity, childhood BMI, gut microbiota, Mendelian randomization, causal relationship

## Abstract

**Background:**

Childhood obesity (CO) is an increasing public health issue. Mounting evidence has shown that gut microbiota (GM) is closely related to CO. However, the causal association needs to be treated with caution due to confounding factors and reverse causation.

**Methods:**

Data were obtained from the Microbiome Genome Consortium for GM as well as the Early Growth Genetics Consortium for childhood obesity and childhood body mass index (CBMI). Inverse variance weighted, maximum likelihood, weighted median, and MR.RAPS methods were applied to examine the causal association. Then replication dataset was used to validate the results and reverse Mendelian randomization analysis was performed to confirm the causal direction. Additionally, sensitivity analyses including Cochran's *Q* statistics, MR-Egger intercept, MR-PRESSO global test, and the leave-one-out analysis were conducted to detect the potential heterogeneity and horizontal pleiotropy.

**Results:**

Our study found suggestive causal relationships between eight bacterial genera and the risk of childhood obesity (five for CO and four for CBMI). After validating the results in the replication dataset, we finally identified three childhood obesity-related GM including the genera *Akkermansia*, *Intestinibacter*, and *Butyricimonas*. Amongst these, the genus *Akkermansia* was both negatively associated with the risk of CO (OR = 0.574; 95% CI: 0.417, 0.789) and CBMI (*β* = −0.172; 95% CI: −0.306, −0.039).

**Conclusions:**

In this study, we employed the MR approach to investigate the causal relationship between GM and CO, and discovered that the genus *Akkermansia* has a protective effect on both childhood obesity and BMI. Our findings may provide a potential strategy for preventing and intervening in CO, while also offering novel insights into the pathogenesis of CO from the perspective of GM.

## Introduction

Currently, childhood obesity has become an increasing public health issue throughout the world (1). The global prevalence of obesity has doubled over the past three decades, affecting over 340 million children (2, 3). Previous studies have demonstrated that childhood obesity is associated with the early onset and development of various chronic diseases, such as cardiovascular disease, asthma, and metabolic syndrome (4, 5). Additionally, there is a significant increase in the risk of mental disorders and mortality in adulthood (6). Despite years of investigation and intervention, the underlying mechanism of childhood obesity (CO) has not yet been fully clarified, and its prevalence continues to rise dramatically.

Gut microbiota (GM) plays a vital role in preserving host physiology and homeostasis. Accumulating evidence has demonstrated a strong association between gut microbiota dysbiosis and two forms of obesity: non-disease-induced obesity and genetic obesity (7–9). However, the majority of human-based studies were designed as observational in nature, while others utilized animal models, resulting in diverse findings across studies. In contrast to previous findings, Schwiertz et al. demonstrated a lower Firmicutes/Bacteroidetes ratio in individuals with obesity (10). Andoh et al. identified *Coprococcus* as a risk factor for obesity (11), while Escobar et al. reported the opposite outcome (12). The observational study design posed challenges in establishing the temporal relationship between exposure and outcome, potentially resulting in reverse causation. Moreover, the results were influenced by confounding factors, including age, dietary patterns, and lifestyle. Lastly, previous research has primarily focused on adults with obesity, while studies involving children are relatively limited. Therefore, caution should be treated when interpreting the association between gut microbiota and childhood obesity.

Mendelian randomization (MR) analysis, which uses genetic variants as instrumental variables (IVs), is a powerful approach to identify and quantify the causal effect of exposure on outcome (13). MR is regarded as the “most natural” randomized controlled trial (RCT) because the alleles from parents to offspring are randomly assigned, freely combined with genotypes remaining stable after birth (14). Its advantages, such as confounding factor minimization and exclusion of reverse causality, make it a valuable tool for causal inference in observational studies. Therefore, our study performed a two-sample bidirectional MR analysis using the genome wide association study (GWAS) summary statistics to explore the causal relationship between GM and CO, which may offer an effective strategy for CO prevention and intervention as well as novel insights to understand the pathogenesis of CO from the perspective of GM.

## Materials and methods

### Data source

The GM dataset was conducted by the Microbiome Genome (MiBioGen) Consortium including 18,340 trans-ancestral subjects (15). After extracting DNA from fecal samples, data were generated by 16S rRNA gene sequencing in the Illumina platform, targeting variable regions of V1–V2, V3–V4, and V4. Setting SILVA as the reference, all the data were annotated to genus and higher levels to profile the microbial composition (16).

GWAS summary statistics for outcomes were extracted from the Early Growth Genetics (EGG) Consortium including childhood obesity and childhood body mass index (BMI). Specifically, CO cases were defined as children whose BMI-for-age were ≥95th percentile at any time from 2 to 18 years old, while controls were individuals whose BMI-for-age were less than the 50th percentile consistently throughout childhood for all available measures. The growth chart and the criteria of childhood obesity was based on the Centers for Disease Control and Prevention in the United States (17). The discovery dataset was collected from a pooled dataset of 30 multiple ancestry cohorts including 28,604 subjects (13,005 cases and 15,599 controls) (18). The replication dataset was obtained from a meta-analysis of 14 studies consisting of 13,848 subjects (5,530 cases and 8,318 controls) (19). For childhood BMI, the phenotype analyzed in both discovery and replication dataset was BMI at the latest time point during childhood, transformed into sex- and age-adjusted standard deviation scores. The discovery dataset consisted of a GWAS meta-analysis of 26 studies, encompassing 39,620 children aged 2–10 years old (20). The replication dataset comprised another GWAS meta-analysis of 20 studies, involving 35,668 children aged 3–10 years old (21). Detailed information on exposure and outcome GWAS datasets was summarized in Supplementary Table S1.

### Instrumental variables

**Figure 1:**
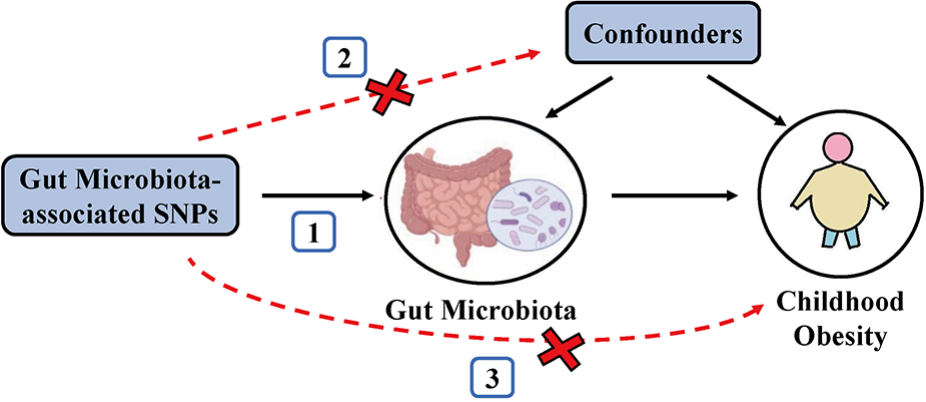
Schematic representation of the MR analysis. The three assumptions of MR are as follows: (1) Instrumental variables must be associated with gut microbiota, (2) instrumental variables must not be associated with confounders; and (3) instrumental variables must influence disease outcomes only through gut microbiota, not through other pathways.

To satisfy the three key assumptions of MR analysis (Figure 1), five steps were applied to select the optimal IVs: (1) SNPs under a locus-wide significance threshold of *P* < 1 × 10^−05^ were obtained as candidate IVs related to GM (22). (2) PLINK clumping method (*r*^2^ < 0.001, clump window <10,000 kb) was performed to ensure the IVs were independent (23). (3) SNPs that are palindromic and those with minor allele frequencies below 0.01 were eliminated. (4) The proxy SNPs (*r*^2^ > 0.8) were selected based on 1,000 Genome project's European population after removing the SNPs closely related to the outcome phenotype (*P* < 5 × 10^−08^) (24). (5) SNPs with *F*-statistics < 10 were excluded to avoid weak IV bias (25).

### Statistical analyses

To detect the causal associations between exposure (GM) and outcomes (CO and CBMI), the inverse-variance weighted (IVW) method was used as the primary MR analysis method. The IVW method, an extension of the Wald estimator for estimating causal effects, constrains the intercept to zero. It calculates the total causal effect using a weighted linear regression model with the weight coefficient (26). IVW results were corrected for multiple comparisons applying the *q*-value procedure (*q* < 0.1), while *P *< 0.05 but *q* > 0.1 was considered to have a suggestive association (27). The analysis was initially performed in the discovery set and subsequently validated in the replication set.

To evaluate the robustness of our study, we also performed several other MR methods including Maximum Likelihood (MaxLik), Weighted Median (WM), and MR robust adjusted profile score (MR.RAPS). The MaxLik method estimates the parameter values by maximizing the likelihood function with small standard errors (28). The WM method increases the power of causal effect when more than 50% of IVs are valid (29). MR.RAPS provides robust estimates to account for systematic and idiosyncratic pleiotropy, even in the presence of weak IVs (30). Reverse MR analysis was employed to confirm the causal direction. It followed similar methods as forward MR, but with CO/CBMI as the exposures and GM as the outcome.

Cochran's IVW *Q* statistics and leave-one-out analysis were employed to evaluate potential heterogeneity. MR-Egger intercept and MR Pleiotropy RESidual Sum and Outlier (MR-PRESSO) global test were conducted to detect whether directional horizontal pleiotropy is driving the results of MR analyses (31, 32).

**Figure 2:**
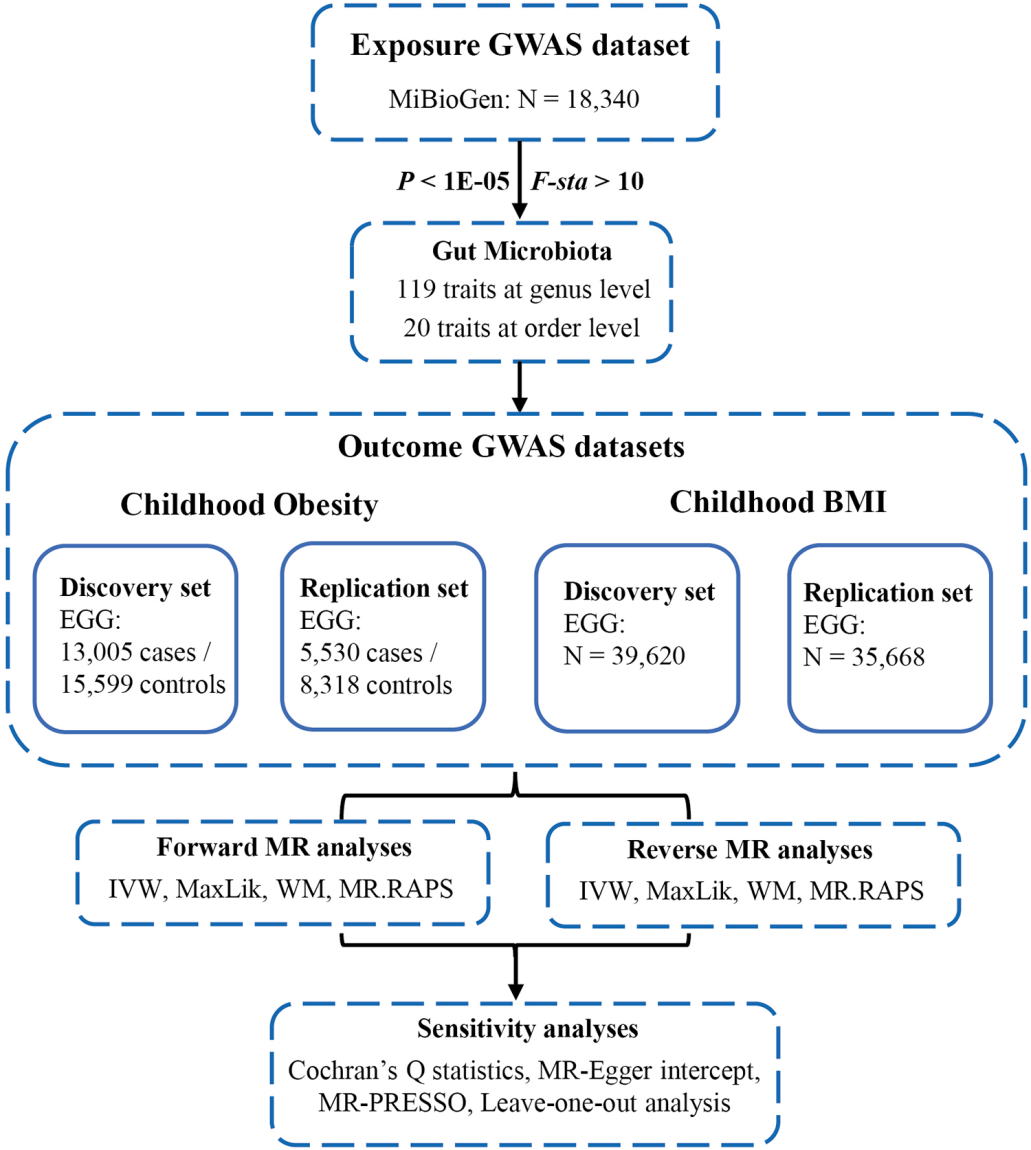
Flowchart of this study. GWAS, genome-wide association studies; MiBioGen, microbiome genome; EGG, early growth genetics; MR, mendelian randomization; IVW, inverse-variance weighted; MaxLik, maximum likelihood; WM, weighted median; MR.RAPS, MR robust adjusted profile score; MR-PRESSO, MR Pleiotropy RESidual Sum and Outlier.

The flowchart of our study was shown in Figure 2. All MR analyses were conducted using the “TwoSampleMR”, “MRPRESSO”, and “qvalue” packages in R software.

## Results

The selected GM instruments based on the criteria containing a total of 8,763 SNPs associated with 119 bacterial genera and 20 bacterial orders. The characters of the selected IVs were presented in Supplementary Table S2.

### Childhood obesity

**Figure 3:**
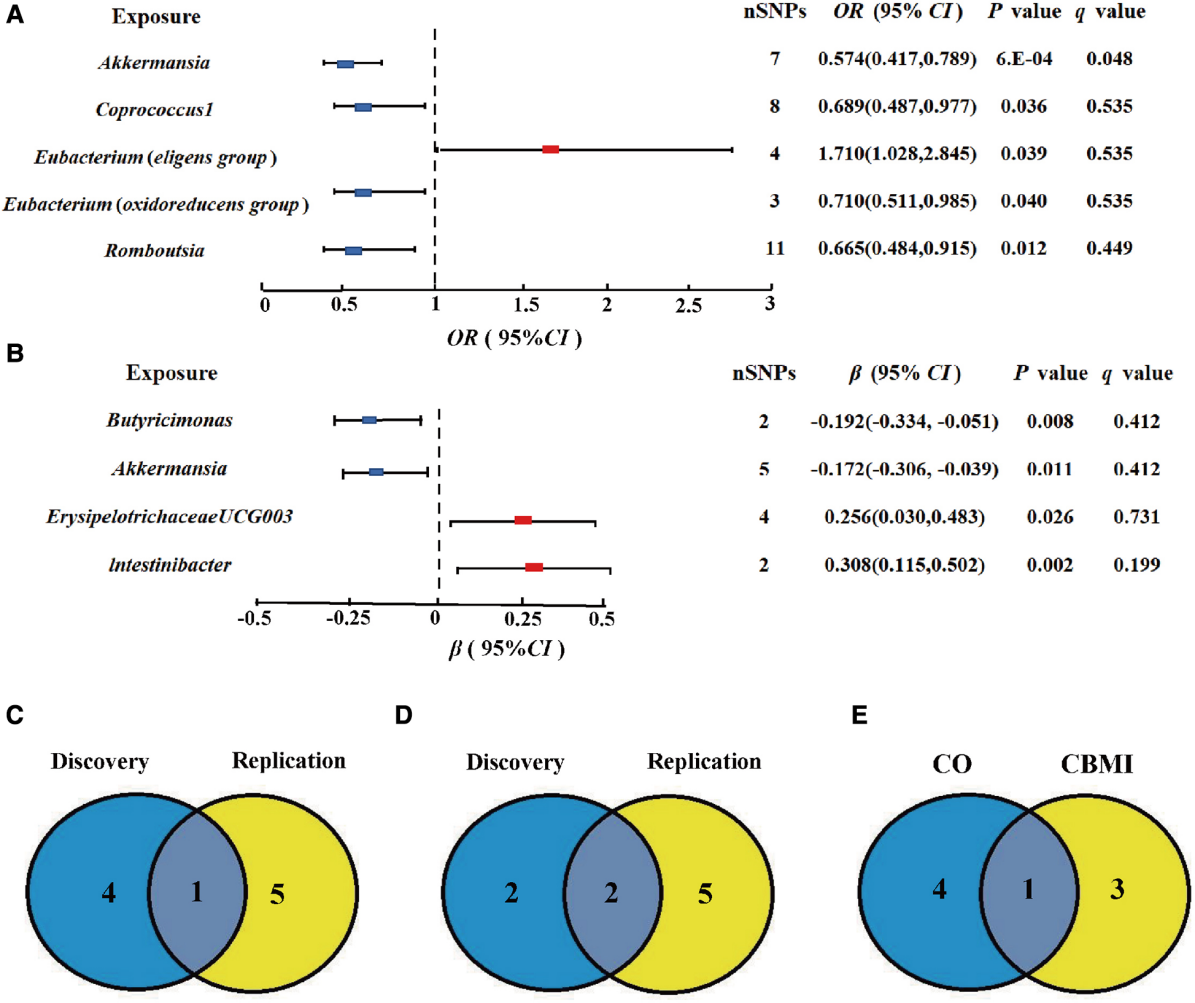
Gut microbiota (GM) which associated with childhood obesity (CO). (**A,B**) Associations of GM with the risk of CO and childhood body mass index (CBMI) using IVW method. (**C**) Venn diagram of the CO-related GM in discovery and replication dataset. (**D**) Venn diagram of the CBMI-related GM in discovery and replication dataset. (**E**) Venn diagram of the causal GM in CO and CBMI dataset SNP, single nucleotide polymorphisms; OR, odds ratio; Cl, confidence interval.

At the genus level, by using the IVW method, we found a suggestive causal association of increase in *Eubacterium* (*eligens group*) (OR = 1.710; 95% CI: 1.028, 2.845; *P* = 0.039) and higher risk of CO, while genetically increased in *Akkermansia* (OR = 0.574; 95% CI: 0.417, 0.789; *P* < 0.001), *Coprococcus1* (OR = 0.689; 95% CI: 0.487, 0.977; *P* = 0.036), *Eubacterium* (*oxidoreducens group*) (OR = 0.710; 95% CI: 0.511, 0.985; *P* = 0.040), and *Roseburia* (OR = 0.665; 95% CI: 0.484, 0.915; *P* = 0.012) were related to protective effects on CO (Figure 3A). After multiple comparison corrected, we still found a significant causal effect of increased *Akkermansia* on the lower risk of CO (*q* = 0.048), the replication dataset also validates this causal relationship (OR = 0.743; 95% CI: 0.586, 0.941; *P* = 0.014; Figure 3C). Details of all the IVW results in discovery and replication datasets were shown in Supplementary Table S3, S4. The *F*-statistics ranged from 21.46 to 25.48 among all the results above. Additionally, causal associations between GM and CO risk were found in more than three MR methods (Table 1, Supplementary Figure S1), including IVW, MaxLik, WM, and MR.RAPS methods. However, at the order level, all the *P* values (ranging from 0.053 to 0.932) were greater than 0.05, indicating no significant associations between bacterial orders and CO (Supplementary Table S5).

**Table 1:** MR analyses of the gut microbiota genera on CO/CBMI by using different methods.

Exposure	Outcome	*F*-stat	*q*	Inverse variance weighted	*P*	Maximum likelihood	*P*	Weighted median	*P*	MR.RAPS	*P*
				OR*/β* (95% CI)		OR*/β* (95% CI)		OR*/β* (95% CI)		OR*/β* (95% CI)	
*Akkermansia*	CO	23.78	0.048	0.574 (0.417, 0.789)	6 × 10^−04^	0.567 (0.406, 0.793)	0.001	0.567 (0.367, 0.876)	0.011	0.641 (0.466, 0.882)	0.006
*Coprococcus1*	CO	25.48	0.535	0.689 (0.487, 0.977)	0.036	0.685 (0.481, 0.976)	0.036	0.668 (0.418, 1.067)	0.091	0.899 (0.650, 1.242)	0.514
*Eubacterium eligens group*	CO	23.03	0.535	1.710 (1.028, 2.845)	0.039	1.715 (1.014, 2.899)	0.044	1.816 (0.999, 3.301)	0.050	1.670 (1.019, 2.737)	0.042
*Eubacterium oxidoreducens group*	CO	21.46	0.535	0.710 (0.511, 0.985)	0.040	0.703 (0.498, 0.992)	0.045	0.644 (0.434, 0.957)	0.030	0.703 (0.490, 1.008)	0.044
*Romboutsia*	CO	25.37	0.449	0.665 (0.484, 0.915)	0.012	0.664 (0.478, 0.922)	0.014	0.756 (0.485, 1.177)	0.215	0.720 (0.527, 0.983)	0.038
*Butyricimonas*	CBMI	25.48	0.412	−0.192 (−0.334, −0.051)	0.008	−0.201 (−0.324, −.0.079)	0.001	−0.197(−0.363, −0.031)	0.020	−0.227(−0.401, −0.053)	0.011
*Akkermansia*	CBMI	27.75	0.412	−0.172(−0.306, −0.039)	0.011	−0.174(−0.313, −0.036)	0.014	−0.195(−0.359, −0.032)	0.019	−0.112(−0.239, 0.015)	0.083
*ErysipelotrichaceaeUCG003*	CBMI	23.48	0.731	0.256 (0.030, 0.483)	0.026	0.254 (0.013, 0.496)	0.039	–	–	0.254(−0.004, 0.513)	0.043
*lntestinibacter*	CBMI	22.53	0.199	0.308 (0.115, 0.502)	0.002	0.312 (0.094, 0.531)	0.005	–	–	0.312(0.083, 0.541)	0.007

The genera *ErysipelotrichaceaeUCG003* and *lntestinibacter* do not have results in weighted median method, because only 2 SNPs were selected as instrumental variables for this two genera and limited SNPs cannot use the weighted median method. CO, childhood obesity; CBMI, childhood body mass index; *F*-stat, *F* statistics to detect weak instrumental variable bias; MR.RAPS, mendelian randomization robust adjusted profile score; OR, odds ratio; CI, confidence interval; *P*, *P* value; *q*, *q* value (adjusted *P* value).

Cochran's *Q* statistics showed no significant heterogeneity in selected IVs (*P* > 0.05 in IVW and MR-Egger methods, Supplementary Table S6). Both the MR-Egger intercept and the MR-PRESSO global test confirmed there is no significant directional horizontal pleiotropy (*P* > 0.05, Supplementary Table S6). Additionally, the leave-one-out analysis revealed that there are no outlier IVs that would have a significant impact on the result if retained (Supplementary Figure S2).

All methods in reverse MR analysis showed no causal relationship from CO to GM (*P* > 0.05, Table 2) except for the genus *Roseburia*. The sensitivity analyses including Cochran's *Q* statistics, MR-Egger intercept, MR-PRESSO global test, and the leave-one-out analysis demonstrated the robustness of the reverse MR results (Supplementary Table S6, Figure S3).

**Table 2:** Reverse MR analyses of CO/CBMI on the gut microbiota genera by using different methods.

Exposure	Outcome	*q*	Inverse variance weighted	*P*	Maximum likelihood	*P*	Weighted median	*P*	MR.RAPS	*P*
			*β* (95% CI)		*β* (95% CI)		*β* (95% CI)		*β* (95% CI)	
CO	*Akkermansia*	0.830	0.027 (−0.067, 0.120)	0.576	0.028 (−0.038, 0.094)	0.412	−0.002 (−0.093, 0.090)	0.973	0.023 (−0.038, 0.084)	0.461
CO	*Coprococcus1*	0.830	−0.010 (−0.065, 0.044)	0.711	−0.010 (−0.065, 0.044)	0.710	−0.024 (−0.091, 0.043)	0.484	−0.069 (−0.122, −0.016)	0.797
CO	*Eubacterium eligens group*	0.830	0.006 (−0.052, 0.065)	0.830	0.007 (−0.052, 0.065)	0.827	−0.010 (−0.085, 0.064)	0.789	−0.005 (−0.060, 0.050)	0.846
CO	*Eubacterium oxidoreducens group*	0.830	−0.042 (−0.138, 0.055)	0.400	−0.042 (−0.139, 0.055)	0.400	−0.049 (−0.163, 0.065)	0.400	−0.033 (−0.127, 0.061)	0.487
CO	*Romboutsia*	0.252	−0.067 (−0.126, −0.007)	0.028	−0.067 (−0.127, −0.007)	0.029	−0.055 (−0.131, 0.021)	0.159	−0.069 (−0.126, −0.012)	0.018
CBMI	*Butyricimonas*	0.453	0.064 (−0.055, 0.183)	0.293	0.065 (−0.055, 0.185)	0.285	0.147 (−0.005, 0.300)	0.059	0.064 (−0.058, 0.186)	0.301
CBMI	*Akkermansia*	0.792	0.036 (−0.087, 0.160)	0.564	0.038 (−0.077, 0.152)	0.521	−0.015 (−0.177, 0.147)	0.858	0.036 (−0.080, 0.152)	0.531
CBMI	*ErysipelotrichaceaeUCG003*	0.792	0.042 (−0.277, 0.362)	0.795	0.044 (−0.150, 0.238)	0.658	0.081 (−0.147, 0.309)	0.488	0.043 (−0.147, 0.233)	0.653
CBMI	*Outcome*	0.453	−0.067 (−0.216, 0.082)	0.380	−0.065 (−0.178, 0.049)	0.263	−0.056 (−0.221, 0.108)	0.502	−0.068 (−0.180, 0.044)	0.233

CO, childhood obesity; CBMI, childhood body mass index; MR.RAPS, mendelian randomization robust adjusted profile score; *CI*, confidence interval; *P*, *P* value; *q*, *q* value (adjusted *P* value).

### Childhood BMI

At the genus level, we found four suggestive causal effects of GM on CBMI (*P *< 0.05, *q* > 0.1) in the discovery dataset. Specifically, *Butyricimonas* (*β* = −0.192; 95% CI: −0.334, −0.051; *P* = 0.008) and *Akkermansia* (*β* = −0.172; 95% CI: −0.306, −0.039; *P* = 0.011) were negatively associated with CBMI, while *ErysipelotrichaceaeUCG003* (*β* = 0.256; 95%CI: 0.030, 0.483; *P* = 0.026) and *lntestinibacter* (*β* = 0.308; 95% CI: 0.115, 0.502; *P* = 0.002) were positively associated with CBMI (Figure 3B). However, after multiple comparison corrected, the causal associations didn't exist but the replication dataset also supported the causal effect of *Butyricimonas* (*β* = −0.101; 95% CI: −0.181, 0.020; *P* = 0.015) and *lntestinibacter* (*β* = 0.191; 95% CI: 0.090, 0.332; *P* = 0.008) on CBMI (Figure 3D). Details of all the IVW results in both discovery and replication datasets were shown in Supplementary Table S7, S8. The *F*-statistics ranged from 22.53 to 27.75 among all the results above. The causal associations between GM and CBMI were found in more than three MR methods (Table 1, Supplementary Figure S4). Furthermore, in the comparison of GM that have a causal relationship with both CO and CBMI, we observed that the genus *Akkermansia* exhibited the protective effect on both outcomes (Figure 3E). However, at the order level, all the *P* values (ranging from 0.074 to 0.996) were above 0.05, indicating no significant associations between bacterial orders and CBMI (Supplementary Table S9).

All methods in reverse MR analysis showed no causal relationship from CBMI to GM (*P* > 0.05, Table 2). The sensitivity analyses demonstrated the robustness of both the forward and reverse MR results (Supplementary Table S10, Figures S5, S6).

## Discussion

In this bidirectional two-sample MR study, we detected suggestive causal associations between eight particular bacterial genera and the risk of childhood obesity (five for CO and four for CBMI). We validated these findings in replication datasets and further identified three gut microbiota (GM) taxa, namely *Akkermansia*, *Intestinibacter*, and *Butyricimonas*, that were associated with childhood obesity. Specifically, our MR analysis revealed a protective effect of *Akkermansia* on childhood obesity (CO) in both the discovery and replication datasets. Importantly, this association remained significant even after correcting for multiple comparisons. Furthermore, this genus was negatively associated with CBMI. *Akkermansia*, a genus in the phylum Verrucomicrobia, can generate acetate and propionate in the human intestinal tract (33). Previous studies have demonstrated an inverse association between the abundance of *Akkermansia* and triglyceride levels as well as BMI in genetically obese mice and mice fed a high-fat diet (34, 35). Karlsson et al. conducted a case-control study involving forty preschool students and observed a reduced concentration of *Akkermansia* in fecal samples from overweight/obese children (36). These findings were consistent with two other cross-sectional studies, providing support for our result (37). Reduced levels of *Akkermansia muciniphila* may lead to excessive intestinal permeability, whereas increased levels of this genus aid in interleukin-36 protection against obesity (38). *Akkermansia* has the potential to prevent simple obesity and exhibit hepatoprotective effects through the inhibition of metabolic pathways involving tyrosine, phenylalanine, and tryptophan (39). Additionally, *Akkermansia* impairs acetyl-CoA oxidation and encourages ketogenesis (40). Taking into account evidence from animal experiments, epidemiological studies, and the potential mechanism by which *Akkermansia* contributes to body weight regulation, several RCTs were conducted to confirm its protective effect (41–43). In a pilot study involving thirty-three overweight/obese subjects, supplementation with *Akkermansia muciniphila* for three months led to reductions in several obesity-related indicators, such as fat mass, hip circumference, and plasma total cholesterol, compared to the baseline data (41). It indicated that the genus *Akkermansia* might be a promising prevention and treatment probiotic target for obesity as well as long-term obesity-related disorders, therefore, further studies could be focused on children to explore its possible effect on childhood obesity.

Our study found that the bacterial genera *Intestinibacter* and *Butyricimonas* were negatively associated with CBMI both in discovery and replication datasets. In a meta-analysis examining GM markers associated with obesity, it was found that twenty-three genera, including *Butyricimonas*, were less abundant in the fecal samples of individuals with simple obesity (44). By inoculating the stools of obesity donors to mice, Rodriguez et al. demonstrated that the *Butyricimonas* contribute to the decrease in adiposity and hepatic steatosis (45). However, further confirmation of its protective effect against the risk of obesity in the pediatric population is necessary. Similar to our findings, Tian et al. reported that a dietary fiber intervention, known for its weight control benefits, significantly inhibits the growth of *Intestinibacter* (46). Another RCT conducted in metformin-treated weight loss people also reported the decreased relative abundance of *Intestinibacter* both in the short and long-time interventions (47). Limited previous studies have investigated the association between *Intestinibacter* and childhood obesity. However, both observational studies and animal models consistently emphasize the role of *Intestinibacter* in the production of butyrate, a metabolite generated through microbial fermentation (48, 49). Butyrate increases fatty acid oxidation in the muscle and decreases lipolysis via the orphan G protein–coupled receptor 43 pathway in white adipose tissue (50). *In vitro* studies demonstrated that butyrate is the primary ATP source for intestinal epithelial cells to absorb nutrition (51). Furthermore, butyrate is metabolized in the liver to produce fatty acids, cholesterol, and ketone bodies, which serve as essential building blocks for fat synthesis (52). In a cross-sectional study, it was observed that the fecal butyrate concentration progressively increases in children with severe obesity when compared to the leaner group (37). All the evidence above may suggest the mechanism that the genus *Intestinibacter* affects body weight by butyrate metabolism, however, further human-based studies and functional experiments are needed to support this association.

Our study has some strengths. Firstly, this is the first MR analysis to investigate the possible causal associations between GM and childhood obesity with the advantages of fewer confounding factors and rare reverse causations. Secondly, we employed two outcomes, namely obesity and BMI, to provide a comprehensive evaluation of childhood obesity. Additionally, we conducted analyses using both discovery and replication datasets to validate our findings. Thirdly, the data sources of exposure and outcome are the largest GWAS to date, along with bidirectional MR and several sensitivity analyses which ensure the robustness of our findings.

There are still several limitations. Firstly, the significance threshold of exposure IVs was set at 1 × 10^−05^ because of insufficient IVs under genome-wide significance. However, we tested the *F*-statistics to exclude the weak instrumental bias. Secondly, the population of the original GWAS is mainly European, which may restrict the generalizability of our findings to other ethnic populations. Thirdly, MR analyses were limited to the order and genus level rather than at a more specific species level due to the constrained resolution of 16S rRNA sequencing. Lastly, we were unable to investigate the relationship between specific measures of general gut microbiota composition and childhood obesity or childhood BMI due to the unavailability of the GWAS dataset on GM composition.

In conclusion, by performing bidirectional MR analyses on GWAS summary data, our study comprehensively explored the causal effects of gut microbiota on childhood obesity. Our findings have the potential to offer valuable insights into the prevention and treatment of childhood obesity, providing a useful strategy, as well as enhancing our understanding of the underlying mechanism from the perspective of gut microbiota. However, further validation of our findings is necessary through additional functional experiments and randomized controlled trials.

## Data Availability

The original contributions presented in the study are included in the article/**Supplementary Material**, further inquiries can be directed to the corresponding author.
