# Supplementary material for: Mendelian randomization analyses support causal relationship between gut microbiota and childhood obesity
Source: Front Pediatr. 2023 Aug 1;11:1229236. doi: 10.3389/fped.2023.1229236 (PMC10427879; doi:10.3389/fped.2023.1229236)
Supplement: Supplementary file 1 [file Datasheet1.docx]

**Supplementary Figures 1-6**

**Mendelian randomization analyses support causal relationship between gut microbiota and childhood obesity**

Figure S1. Scatter plots for the causal relationship between gut microbiota and childhood obesity.

Figure S2. Leave-one-out plots for the causal association between gut microbiota and childhood obesity in forward MR analyses.

Figure S3. Leave-one-out plots for the causal association between gut microbiota and childhood obesity in reverse MR analyses.

Figure S4. Scatter plots for the causal relationship between gut microbiota and childhood BMI.

Figure S5. Leave-one-out plots for the causal association between gut microbiota and childhood BMI in MR analyses.

Figure S6. Leave-one-out plots for the causal association between gut microbiota and childhood BMI in reverse MR analyses.

**
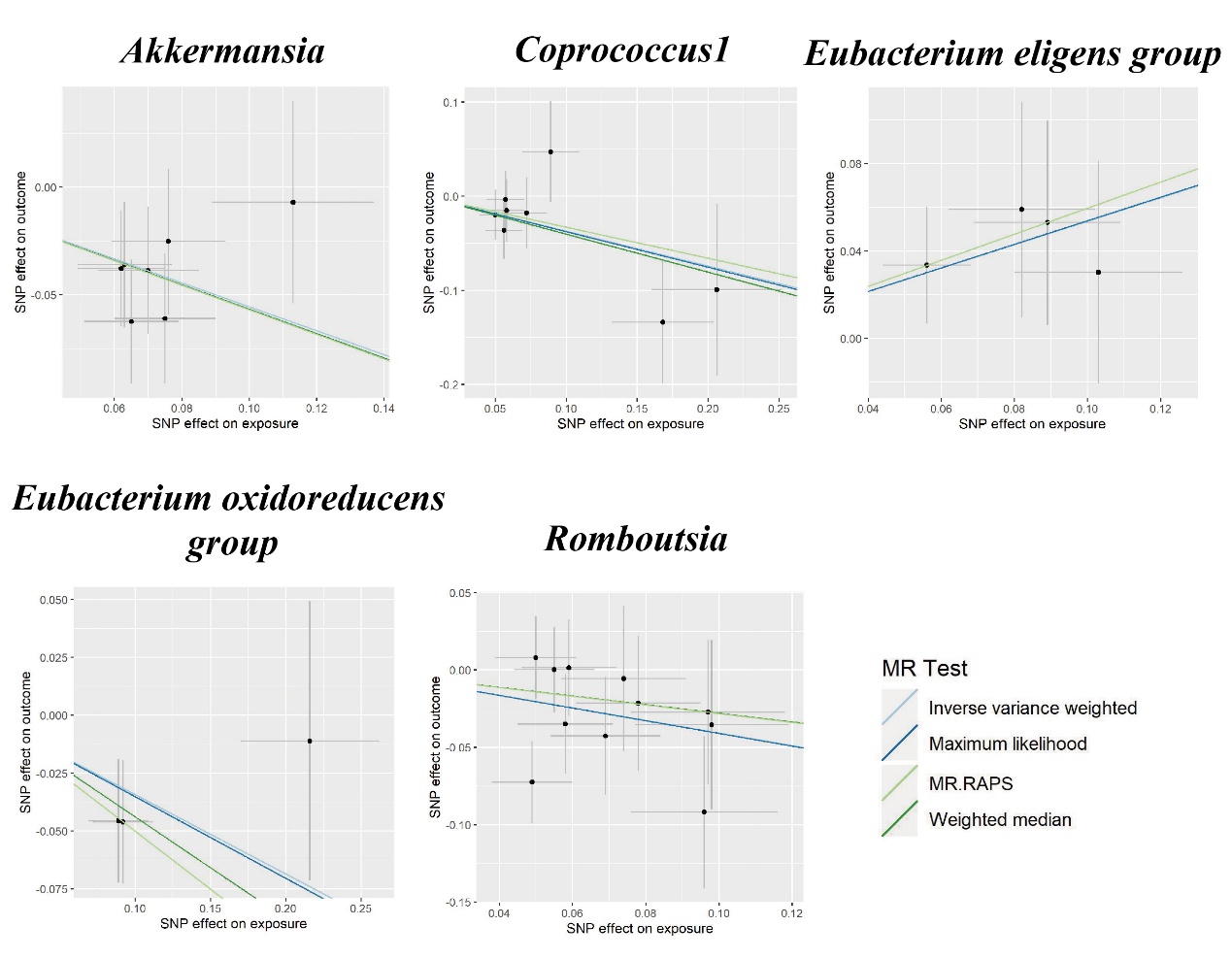
**

**Figure S1.** Scatter plots for the causal relationship between gut microbiota and childhood obesity.

**
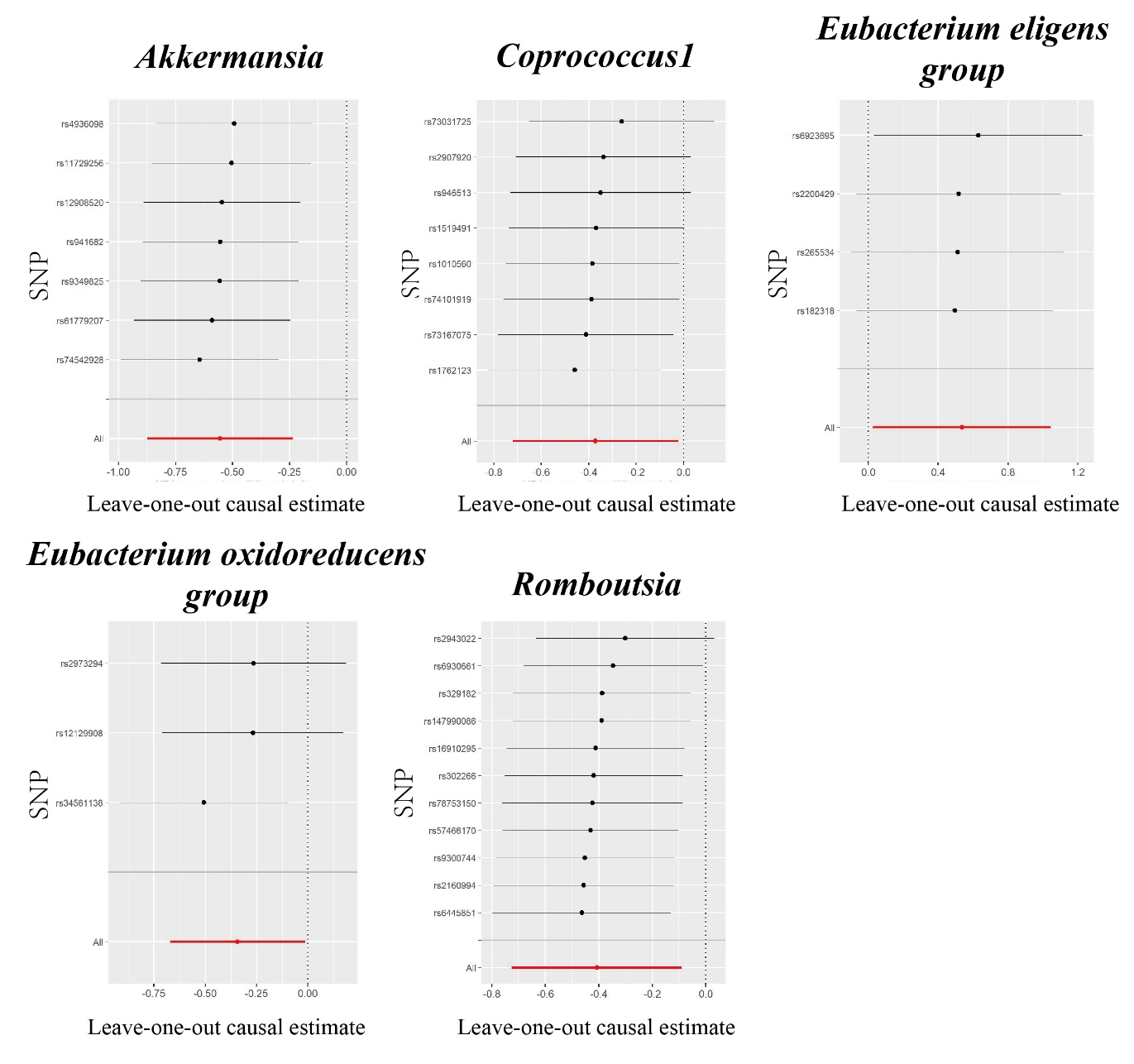
**

**Figure S2.** Leave-one-out plots for the causal association between gut microbiota and childhood obesity in forward MR analyses.


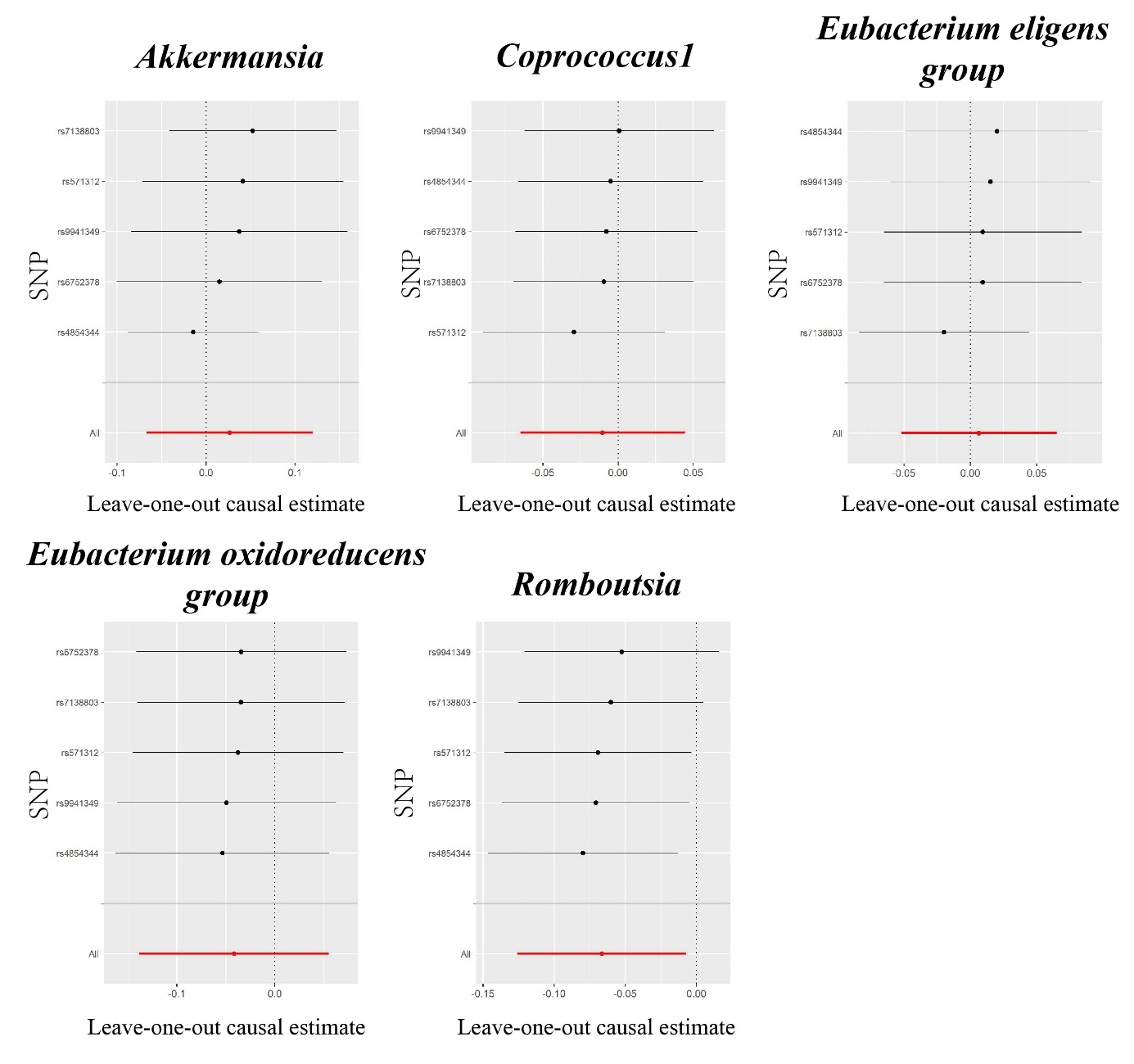


**Figure S3.** Leave-one-out plots for the causal association between gut microbiota and childhood obesity in reverse MR analyses.


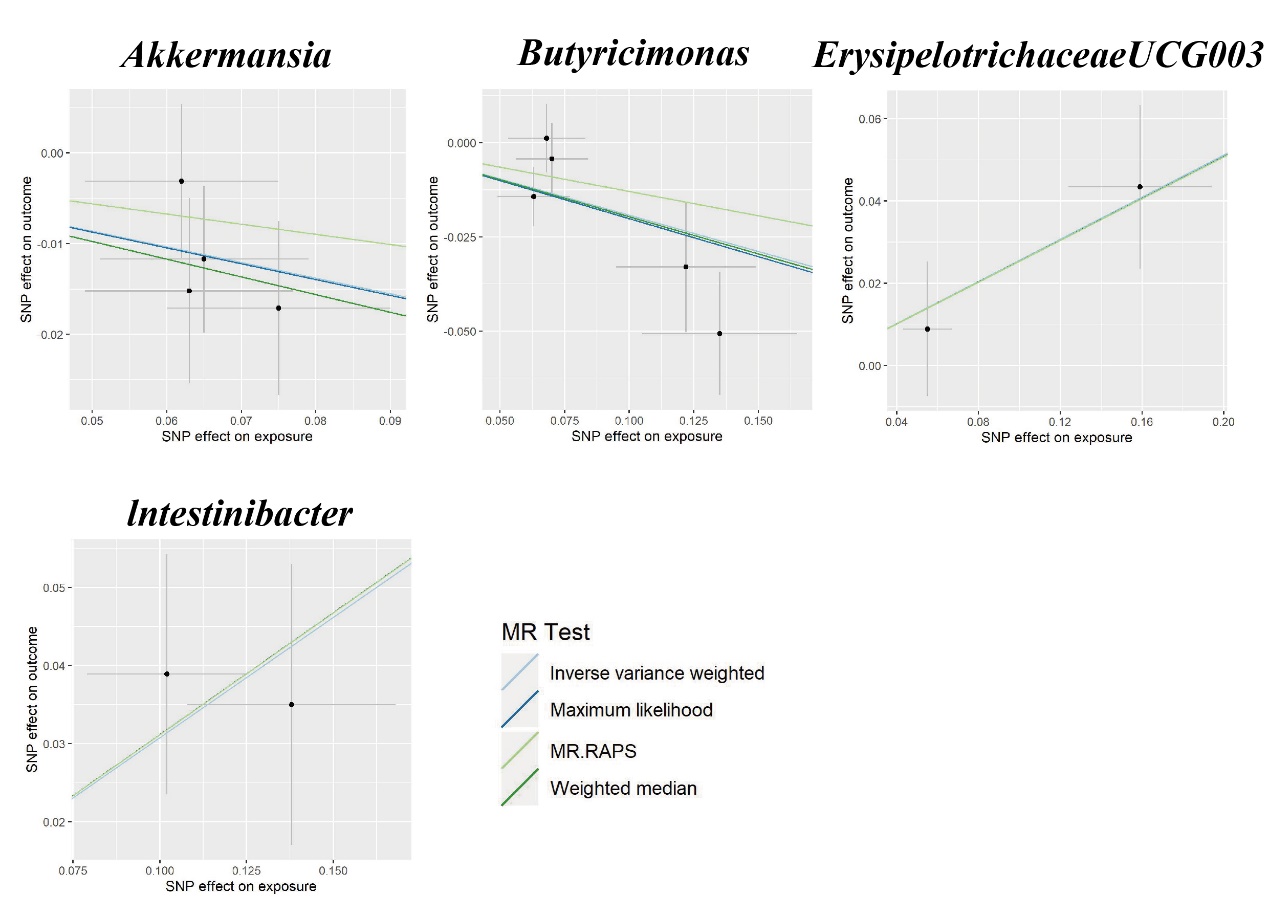


**Figure S4.** Scatter plots for the causal relationship between gut microbiota and childhood BMI.

**
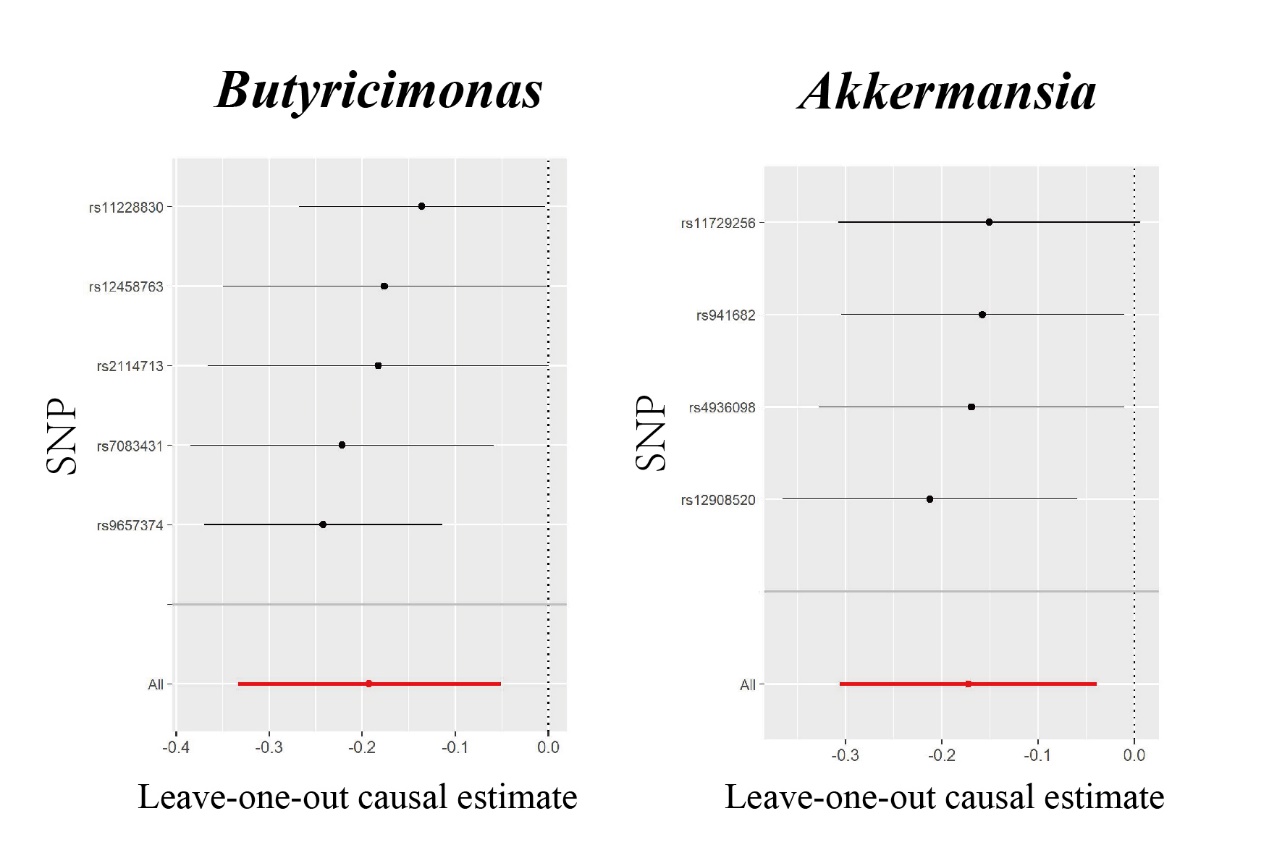
Figure S5.** Leave-one-out plots for the causal association between gut microbiota and childhood BMI in MR analyses.


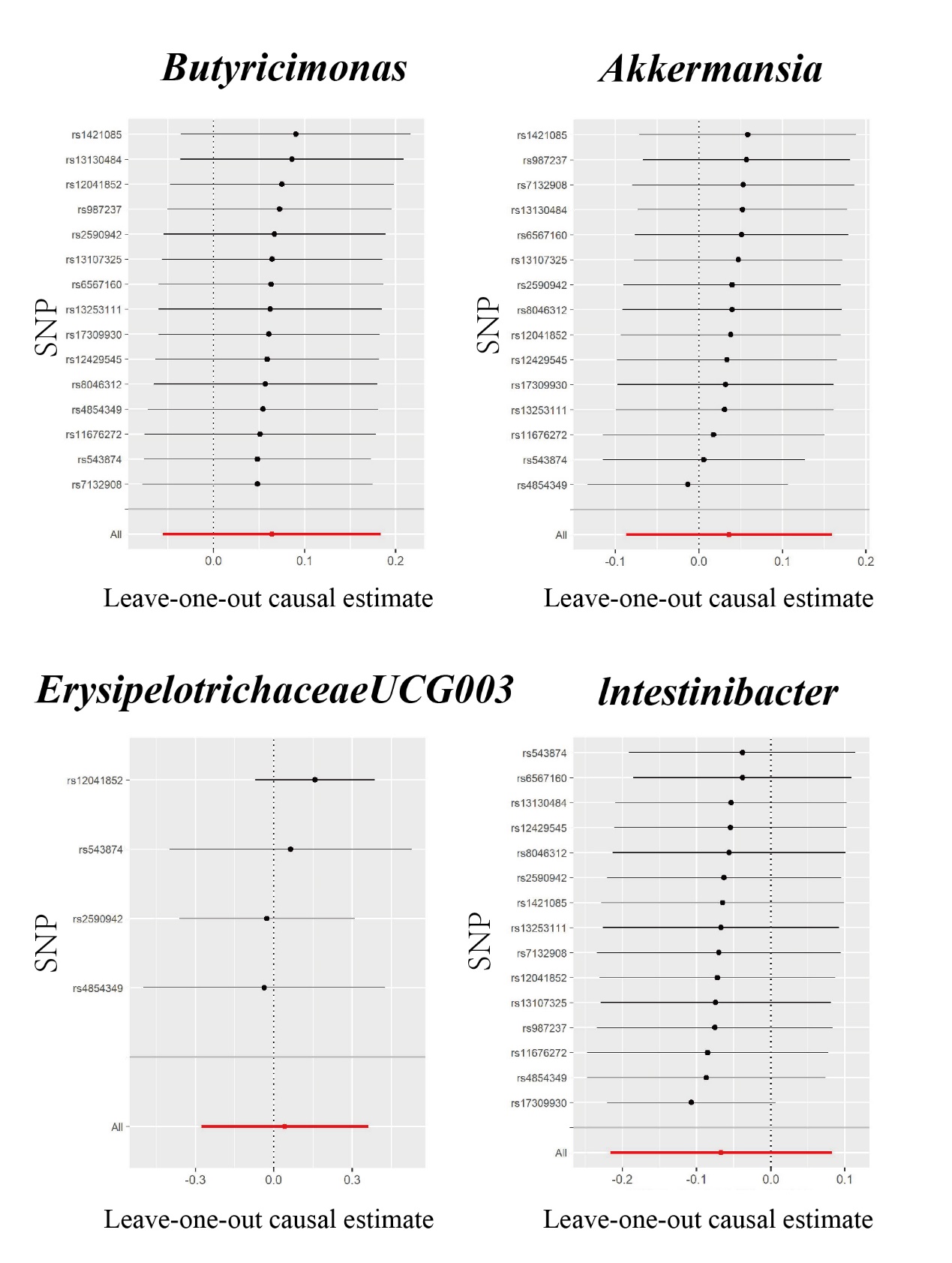


**Figure S6.** Leave-one-out plots for the causal association between gut microbiota and childhood BMI in reverse MR analyses.
